# Camouflage predicts survival in ground-nesting birds

**DOI:** 10.1038/srep19966

**Published:** 2016-01-29

**Authors:** Jolyon Troscianko, Jared Wilson-Aggarwal, Martin Stevens, Claire N. Spottiswoode

**Affiliations:** 1 University of Exeter, Centre for Ecology and Conservation, College of Life & Environmental Sciences, Penryn Campus, Penryn, Cornwall, TR10 9FE, UK; 2 University of Cambridge, Department of Zoology, Downing Street, Cambridge CB2 3EJ, UK; 3 DST-NRF Centre of Excellence at the Percy FitzPatrick Institute, University of Cape Town, Rondebosch 7701, South Africa

## Abstract

Evading detection by predators is crucial for survival. Camouflage is therefore a widespread adaptation, but despite substantial research effort our understanding of different camouflage strategies has relied predominantly on artificial systems and on experiments disregarding how camouflage is perceived by predators. Here we show for the first time in a natural system, that survival probability of wild animals is directly related to their level of camouflage as perceived by the visual systems of their main predators. Ground-nesting plovers and coursers flee as threats approach, and their clutches were more likely to survive when their egg contrast matched their surrounds. In nightjars – which remain motionless as threats approach – clutch survival depended on plumage pattern matching between the incubating bird and its surrounds. Our findings highlight the importance of pattern and luminance based camouflage properties, and the effectiveness of modern techniques in capturing the adaptive properties of visual phenotypes.

The impact of camouflage on survival has been an active area of research since the seminal texts of Wallace^1^ and Cott^2^, but has been especially resurgent in the last decade. Numerous studies have tested specific visual mechanisms that make camouflage effective^3^,^4^,^5^, but these have typically either been laboratory-based, or used artificial model or computer-simulated prey. It has proven far more difficult to quantify and test camouflage in real-world conditions, and thus directly determine the survival advantage that it confers. Kettlewell’s classic experiments during the 1950s demonstrated that dark, melanic morphs of the peppered moth *Biston betularia* were more likely to be recaptured when released in areas with trees blackened by industrial pollution, and further experiments implicated birds as the selective agent underlying the spread of melanic forms^6^. While this revealed a correlation between coloration and survival, neither Kettlewell’s experiments nor numerous subsequent studies^7^,^8^ have either quantified peppered moth camouflage to predator vision, or shown how each individual’s level of background matching directly predicted its survival. In fact, to our knowledge, this has never been done for any species.

Here we demonstrate that the survival of ground-nesting birds’ clutches in Zambia is directly linked to their degree of background matching camouflage, as modelled through multiple predator visual systems. Ground-nesting birds are an ideal study system because the background against which their camouflage functions is unambiguous, because their nests and surroundings remain largely visually constant throughout incubation, and because in the absence of a nest structure, they rely heavily on camouflage to avoid predation. We chose highly cryptic focal species that made little or no modification to the nest surrounds, and used a range of species such that we could assess general principles of camouflage that are not limited to just one species and background type. We studied three nightjar species (Caprimulgiformes, Caprimulgidae: fiery-necked nightjar *Caprimulgus pectoralis* n = 43, Mozambique nightjar *Caprimulgus fossii* n = 36, and pennant-winged nightjar *Macrodipteryx vexillaria* n = 9), three plover species (Charadriiformes, Charadriidae: crowned plover *Vanellus coronatus* n = 37, wattled plover *Vanellus senegallus* n = 6, and three-banded plover *Charadrius tricollaris* n = 3), and three courser species (Charadriiformes, Glareolidae: bronze-winged courser *Rhinoptilus chalcopterus* n = 22, Temminck’s courser *Cursorius temminckii* n = 8, and three-banded courser *Rhinoptilus cinctus* n = 3) in southern Zambia. Camouflage was quantified in terms of luminance, pattern and colour metrics based on their main predator’s visual systems using calibrated digital image analysis^9^.

## Results

**Figure 1:**
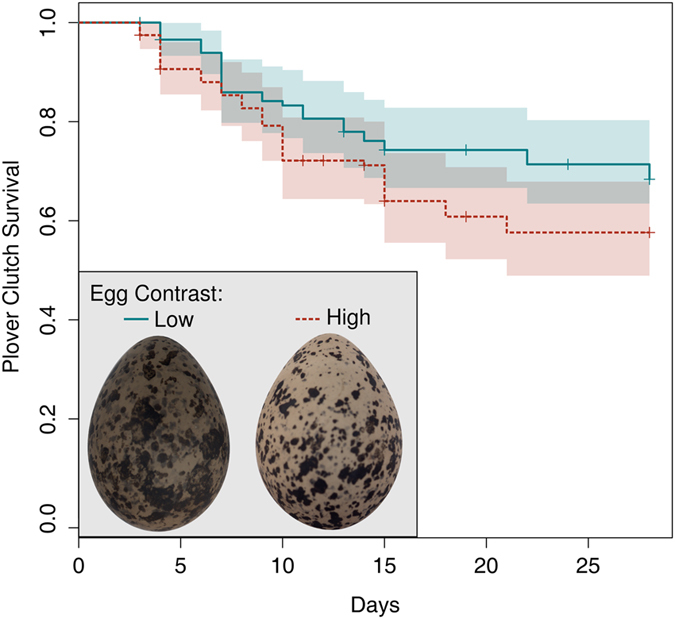
High contrast plover eggs were less likely to survive than low contrast eggs. Eggs in this plot were classified as being high or low contrast if they were above or below the population median respectively, and shaded areas show 95% confidence intervals. The egg examples shown are the lowest (left) and highest (right) contrast crowned plover eggs.

**Figure 2:**
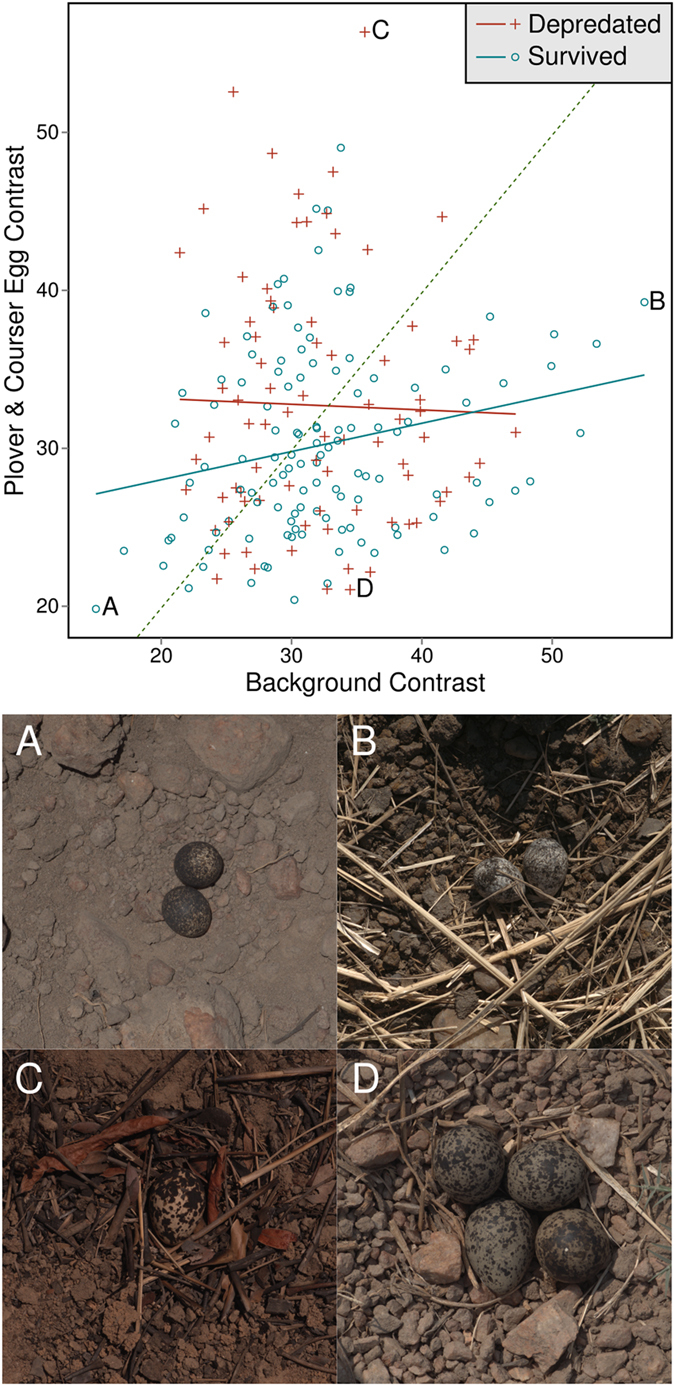
Egg contrast predicted likelihood of plover and courser egg survival, dependent on background contrast. Clutches that survived to hatching better matched their backgrounds with respect to contrast, than those that were depredated. The dashed identity line shows where egg contrast would match background contrast. Panels **(A–D)** give extreme examples of contrast matching, with corresponding points labelled on the graph: (**A**) Temminck’s courser with the lowest background contrast and low egg contrast (note that this egg is a poor luminance match, which was not found to predict clutch survival); (**B**) three-banded plover with the highest background contrast and high egg contrast; (**C**) bronze-winged courser with the highest egg contrast and medium background contrast; (**D**) wattled plover with the lowest egg contrast of all depredated nests, and medium background contrast.

Adult plovers and coursers fled from their nests at long range (61.89 m ± 37.61, mean ±1 standard deviation Table 1), indicating that the main selection pressure is on the appearance of eggs rather than adults. Clutch survival was greater at those nests where the absolute degree of egg contrast was lower (survival analysis, 27 predation events, 79 nests: Z = 2.41; *p* = 0.016; Fig. 1). Moreover, there was a significantly steeper positive correlation between egg contrast and background contrast in clutches that survived, than in clutches that were depredated (generalised linear mixed model, GLMM hereafter, 67 nests: *F*_1, 118_ = 12.30; *p* = 0.001; Fig. 2): this reveals that clutches with low contrast eggs were more likely survive on low contrast backgrounds, and clutches with high contrast eggs were more likely to survive on high contrast backgrounds. Thus, where there was a mismatch between egg contrast and background contrast the clutch was less likely to survive.

**Figure 3:**
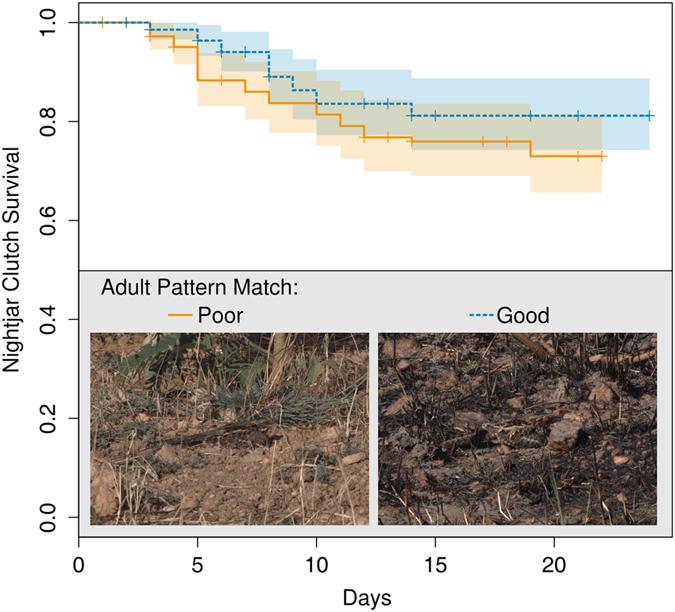
Nightjar adults that better matched the patterns of their backgrounds during incubation were more likely to have their clutch survive until hatching that those that were a poorer match. Nightjars in this plot were classified as being above or below the median pattern difference value (good and poor pattern match respectively), and shaded areas show 95% confidence intervals. Examples shown were the worst (left) and best (right) Mozambique nightjars at matching their background pattern.

Adult nightjars fled from their nests at close range (1.91 m ± 1.38, Table 1), indicating that selection for camouflage should act mainly on the appearance of the incubating adult rather than the eggs. We found that clutch survival was higher at those nests where the plumage of the incubating adult was a better match to its background with respect both to pattern (survival analysis: 19 predation events, 82 nests: Z = −2.58; *p* = 0.001; Fig. 3), and to contrast (GLMM with abandoned and censored nests excluded, leaving 61 nests; surviving clutches had a significantly more positive correlation between adult contrast and background contrast than depredated clutches: *F*_1, 109_ = 8.74; *p* = 0.004). Clutch survival was unrelated to any aspect of egg appearance (no models were a better fit than the null containing only random effects, survival model 85 nests, GLMM 65 nests, *p* > 0.05), supporting our hypothesis that adult plumage provides nightjars with their primary defence against detection.

## Discussion

To our knowledge these are the first data to demonstrate a clear link between the survival of individual wild animals in a natural system and their level of camouflage to predator vision. While other studies have found associations between camouflage and survival in natural systems, they have been unable to quantify camouflage appropriately with respect to the visual systems of the relevant predators. For example, stone curlew *Burhinus oedicnemus* clutches were more likely to survive to hatching if their egg colour matched the background colour^10^. However, colour match in this case was judged using human vision, which often differs substantially from that of the most relevant predator species. Black-tailed gull *Larus crassirostris* clutches were more likely to survive when the eggs had a better colour match to their background^11^, but images of the nests were not appropriately calibrated^9^,^12^, disregarded ultraviolet wavelengths (which many predators can see), and were not modelled through predator vision. Colour matching has been the main focus of the effectiveness of camouflage, rather than pattern or luminance^13^,^14^,^15^,^16^,^17^. Contrary to these findings, we did not find that degree of colour matching was a significant predictor of clutch survival, perhaps because the background colour match was generally good in the natural system we studied (Table 2). Instead, we found that the degree of background pattern matching of the incubating adult was the best predictor of clutch survival in nightjars. This supports previous field studies that measured pattern matching indirectly, for example those finding that artificially-presented spotted eggs suffered fewer avian detections than plain eggs^18^, and work showing that western snowy plover *Charadrius alexandrinus nivosus* nests were more likely to survive when more egg-sized stones were nearby^19^. However, stone size in that study could have been associated with different habitats and predator communities rather than visual appearance alone. We also found that high contrast plover and courser eggs suffered higher predation overall, unless they were laid on high contrast backgrounds. Likewise, incubating nightjars were less likely to suffer clutch predation when their plumage contrast matched the background contrast, compared to when it was a poor match. High contrast patterns are known to be adaptive for improving edge disruption^4^. However, recent work has also shown that humans learn to find high contrast prey faster than low contrast prey^20^, suggesting that high contrast camouflage is likely to be most effective where opportunities for predator learning are low, as may be likely in this diverse prey community with generalist predators. Our study is the first to verify a long-standing assumption that a free-living animal’s camouflage – as modelled through a predator’s eyes – protects it from predation. Future work should seek to further test camouflage in other taxa and varied environments to understand and quantify the adaptive advantages of camouflage, particularly with respect to its under-studied pattern and luminance components which this study suggest to be crucial in natural systems. Finally, our study underlines how camouflage is the product of both prey ecology and the visual systems of the appropriate predators. We need not only to discover more about what makes effective camouflage in real-world situations, but also about how visual systems have evolved to break it.

## Methods

### Field Work

We carried out fieldwork within an area of *c*. 3100 ha around Musumanene and Semahwa farms (centred on 16°46′S, 26°54′E), and *c*. 400 ha on Muckleneuk farm (centred on 16°39′S, 27°00′E), all in the Choma District, Zambia, during September–November 2012–2013. This corresponds to the hot dry season when there is an open under-storey providing nesting habitat for ground-nesting birds. The habitat is a mix of grassland, deciduous miombo woodland and agricultural land (maize, tobacco, ploughed and fallow fields), such that predator communities are likely to approximate historical conditions prior to human disturbance. Nests were principally located by local farm labourers as the birds fled on approach of the searchers or their cattle, and some nightjars were located through nocturnal eye-shine. Thus, although we may have overlooked some of the most-camouflaged plover and courser nests, and may not have found nests with the worst camouflage because predators found them first, there is no reason to expect this to introduce any systematic survival bias and our results nonetheless still show an effect of camouflage on survival.

Clutch survival to hatching was assessed through regular checking every second day. Nightjar chicks did not move from the nest site more than *c.* 1 m in the first 48 hours, so reliably indicated hatching success. Plover and courser clutch survival was judged based on any evidence of predator activity (such as footprints, crunched eggshell and disappearance of eggs before the end of incubation); if the incidence or cause of disappearance could not be ascertained (i.e. eggs were missing with no signs of either hatching or predation), the data were “censored” at their last known date of existence (15 nests)^4^. Note that in survival analysis, censored data are used by the model up until the point of censoring, making them informative even when the nest is destroyed by events not related to predator activity. Further nests were censored due to other incidents not related to predator activity, i.e. trampling by cattle (four nests), ending the field season (three nests), human disturbance (two nests), termite activity (one nest), and bush fire (one nest). Nests were also censored if eggs remained present but deserted by the incubating parents. Deserted nests were identified from an absence of incubating adults over two or more successive visits combined with egg temperatures that matched the environment, such as hot-to-touch in the sun, or cold in the early morning (31 nests; the causes of desertion were unknown, but possibly included human disturbance near footpaths and fields, infertile or heat-damaged eggs, or adult mortality).

Camera traps were placed at a subset of nests to identify predator visual systems and predation events. Footprints also revealed a likely mongoose (unknown species) predation event, and the presence of maize husks stolen from nearby fields suggested a yellow baboon (*Papio cynocephalus*) predation event. Following previous studies of flight initiation distance^21^, distances at which incubating birds fled their nests were recorded whenever possible on approaching the nests in full view of the incubating adult. Nests were approached at a steady speed and distances below *circa* 20 m were measured by pacing, while larger distances were measured by GPS.

### Photography

Adult nightjars incubating their clutch were photographed from a distance of 5 m with the camera angled towards their most visible flank. If both flanks were unobstructed, we chose the side that avoided photographing directly towards the sun. Diurnal incubation is carried out largely (Mozambique nightjar) or exclusively (fiery-necked and pennant-winged nightars) by the female^22^, such that photos taken at a single time point are representative of what predators encountered throughout incubation. Nightjar, plover and courser clutches were photographed *in situ* from 1.25 m directly overhead, and then again under controlled lighting conditions (eggs shaded from direct sunlight and photographed against a uniform background next to the grey standard). All photographs were taken with a Nikon D7000 (fitted with a 105 mm Micro-Nikkor lens, which transmits UV) converted to full spectrum sensitivity by removal of its UV and IR blocking filter (Advanced Camera Services Limited, Norfolk, UK), replacing it with a quartz sheet to allow quantification of colour throughout the avian visible spectrum^9^. Human-visible spectrum photographs were taken through a Baader UV-IR blocking filter (Baader Planetarium, Mammendorf, Germany), permitting only visible spectrum light from 420 to 680 nm, and UV photographs were taken with a Baader UV pass filter permitting ultraviolet light from 320 to 380 nm. All photographs were taken at f/8, ISO400, in RAW format, not within 2 hours of sunrise or sunset, and only in direct sunlight, because this is the most representative natural illumination regime in the Zambian dry season. For the analysis of adult nightjar camouflage, photographs of nightjars incubating their clutch were taken at a distance of 5 m. Once the adult nightjar fled its nest, a 40% Spectralon grey standard (Labsphere) was photographed beside its eggs from 2 m using identical camera settings (a sequential calibration method^9^,^23^). Linearisation curves, used to correct the non-linear relationship most cameras have between light intensity and image pixel values^9^,^12^, were modelled from eight calibrated Spectralon reflectance standards from 99 to 2% reflectance (Labsphere), and linearisation curves for all channels had R^2^ values ≥0.999. Visible and UV photographs were automatically aligned and scaled (to account for camera movement and focal length changes when re-focusing in UV), using customized code that saved 16-bit TIFF images with red, green and blue channels from the human-visible spectrum, and the red and blue channels photographed through the UV pass filter (the green channel has very low UV sensitivity and was discarded)^9^. To avoid saturation, where reflectance values would be greater than 100%, all images were scaled to preserve the highest pixel value. For example, if the highest pixel value represented 120% reflectance, all 16-bit image values were scaled by 1/1.2, and then prior to processing they were scaled back up in 32-bit floating point images to eliminate pixel saturation^9^. Adult nightjar outlines were selected using the freehand selection tool, and eggs were selected with an egg-shape selection tool^24^. Any objects obstructing the *in situ* targets (such as blades of grass and their shadows) were selected out, preventing any ambiguous sections of the target or background from being measured.

### Quantifying Camouflage

We used camera traps to identify biologically relevant predators. This revealed a broad range of diurnal predators, comprising dichromats (one incident of banded mongoose *Mungos mungo*), trichromats (one incident of vervet monkey *Chlorocebus pygerythrus* and one human), and tetrachromats (two incidents of grey-headed bushshrike *Malaconotus blanchoti*) (Movie 1). These predator groups are in line with previously reported predator groups for fiery-necked nightjars, Mozambique nightjars, and crowned plovers^22^. Identification of these predator groups allowed us to map digital images to corresponding models of predator vision^9^,^12^,^23^,^25^,^26^,^27^,^28^ using the most phylogenetically relevant model visual systems available. These were ferret *Mustela putorius furo*, human^29^, and common peafowl *Pavo cristatus*^30^ respectively. We generated models of how a predator would perceive each scene by comparing the predicted camera response and predator cone-catch quanta to thousands of natural reflectance spectra, and then generated polynomial models that mapped from camera to predator cone-catch quanta^9^, producing 32-bits per channel floating point images that overcome problems of saturation (see above). The mapping functions for this camera converting to cone-catch quanta were very accurate for the natural spectra dataset they were generated from (R^2^ values across all receptor channels ≥0.998). Similarly, the cone-catch mapping errors for colour chart values compared to spectrometer measurements were low (R^2^ values across all receptor channels ≥0.966)^9^. We calculated both absolute measures of the appearance of the eggs, adult bird and nest surroundings, and relative measures which quantified the degree of background matching between eggs or adult birds and their surroundings. Each measurement was calculated separately for each predator visual system. Absolute measures were (i) mean luminance (perceived lightness), and (ii) mean contrast (the standard deviation of luminance). Relative measures were (i) luminance difference, (ii) pattern difference, and (iii) colour difference.

Camouflage in adult nightjars was quantified from the differences between the *in situ* bird and its surroundings in the same cone-catch image. Camouflage in eggs of all species was quantified from the difference between the eggs photographed under controlled conditions and their *in situ* clutch surroundings (excluding the *in situ* eggs). Egg images taken under controlled lighting conditions were re-sized using bilinear interpolation to match the pixels/mm of the *in situ* surrounds. Pattern, luminance and contrast metrics were based on luminance-channel images (as with past work^31^) because pattern is widely thought to be primarily encoded by achromatic vision^32^. Ferret luminance was taken to be the L cone sensitivity (L-cones outnumber S-cones 14:1^33^). Human luminance was taken as (L + M)/2^25^, and peafowl as double cone sensitivity^34^. Luminance distribution differences (Luminance_diff_) were calculated by comparing absolute differences in counts of the numbers of pixels in each target (plover egg or adult nightjar plumage) to its background at 32 linear levels of luminance from 0% to 100%:


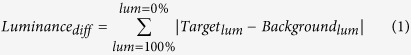


Luminance_diff_ values describe to what extent the egg or nightjar reflectance values, as perceived by a given predator, matched the values of their surrounds. Pattern differences were generated using Fast Fourier Transform bandpass filters at 17 levels (from 2 pixels, increasing exponentially with √2 to 512 pixels), using the standard deviation of luminance values at each spatial scale to represent the ‘energy’ at that spatial scale. Fourier analysis and bandpass filtering have been used in a number of previous studies to analyse animal markings^31^,^35^,^36^. Spatial frequency differences (Pattern_diff_)^9^ were calculated in a similar manner to Luminance_diff_, by summing the absolute differences in energy between target and background at each spatial scale *s* :


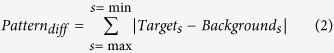


Any differences in pattern energy between the samples at any spatial scale will increase the Pattern_diff_ value. Thus Pattern_diff_ describes the degree to which egg and plumage patterns match the patterns in their surrounds with respect to size, spacing and contrast. When comparing two patterns, this approach has a number of advantages over previous methodologies that separate out the energy spectra into multiple descriptive statistics^9^,^31^,^35^. For example, spatial energy spectra can often be complex and multi-modal, so selecting only the peak frequency or peak energy discards much of that potentially important pattern information at other scales, and can arbitrarily switch between peaks in a multi-modal distribution. Combining pattern similarity into a single measure also makes statistical analysis more straightforward, and has been used for comparing eggs and plumage differences^37^. Pattern_diff_ tests whether the contrast of irregular patterns in the target matches the contrast of irregular patterns in the background at a given spatial scale, disregarding phase information. Therefore, it tests a general background-matching hypothesis rather than a template-matching hypothesis. The latter would be more appropriate for testing for the existence of masquerade, where we would expect the target to be misclassified as a common background object^5^.

Contrast was taken as the standard deviation of luminance pixel values following a square-root transform (to create a normal distribution of luminance values). Likewise, mean luminance was based on square-root transformed luminance values.

Colour analysis was based on the Vorobyev and Osorio^38^ noise model of colour discrimination, generating “just noticeable differences” (JNDs) between colours. Weber fractions were calculated from visual system-specific cone ratios (shortest to longest wavelength; ferret 1 : 14^33^; human 1 : 5.49 : 10.99^39^; peafowl 1 : 1.9 : 2.2 : 2.1^34^). A noise-to-signal ratio of 0.05 was used for the most abundant cone type in each species. In order to determine the most common colours in a scene for each visual system, a local and global colour matching script was used. This script classified any area as a single colour if adjacent pixels were within a 0.05 JND (local) threshold, allowing smooth gradients of colour to be clumped together. The script then searched through the image for any other pixels within a 1 JND (global) threshold, linked these areas, and continued until no more pixels of the same colour were found. The relative cone catch ratios and image coverage for each colour were recorded until 99% of the image was covered, or the 32 most abundant colours were found. Colour difference for adult nightjars was the mean difference (in JNDs) between the most abundant colour in the adult nightjar and all the colours found in its surrounds, weighted by coverage. Likewise, the most abundant colour in plover eggs was compared to the colours in its surroundings.

### Statistics

Statistics were performed in R version 3.2.2^40^ Camouflage metrics were all continuous variables, transformed where necessary to ensure that residuals fitted a normal error distribution. Survival was modelled using mixed-effects Cox proportional hazards with stepwise model simplification of a maximal model containing all camouflage variables. This methodology allows the inclusion of ‘censored’ data, i.e. clutches that survived for an observed period of time even if the outcome of the nest was uncertain^4^,^41^; however, these models were not able to fit higher level interactions. Therefore, interaction effects between absolute camouflage variables were modelled in linear mixed models that could converge even when complex, unlike mixed-effects survival models, but cannot handle censored data. Species identity was included in all maximal models, and nest identity was included as a random factor given the repeated measures generated for each predator visual system. When approached by a simulated predator (ourselves) at consistent speed, nightjars fled from their nests at much shorter distances than plovers and coursers (above, Table 1). We therefore analysed them separately, predicting that adult camouflage should be more important than egg camouflage for nightjar clutch survival. Note that our hypothesis makes a prediction after species differences have been accounted for, rather than a comparative hypothesis that would be confounded with habitat and phylogenetic differences. For example, we are able to ascertain whether having a given camouflage difference relative to each individual’s background makes it more or less likely to survive after the differences in survival and nesting habitat between species are accounted for.

## Additional Information

**How to cite this article**: Troscianko, J. *et al.* Camouflage predicts survival in ground-nesting birds. *Sci. Rep.* **6**, 19966; doi: 10.1038/srep19966 (2016).

## Supplementary Material

Supplementary Movie 1

## Figures and Tables

**Table 1:** Summary statistics of the first recorded distance at which an incubating bird fled its nest.

Species	n	Mean	Min	Max	SD
Mozambique nightjar	32	1.68	0.5	4	0.85
fiery-necked nightjar	41	2.08	0.5	10	1.71
pennant-winged nightjar	5	1.94	1.2	4	1.18
**nightjar totals**	78	1.91	0.5	10	1.38
three-banded plover	4	39	26	56	13.71
Temminck's courser	8	54.63	33	77	12.69
bronze-winged courser	14	42.36	9	77	17.21
three-banded courser	3	8	4	15	6.08
crowned plover	24	84.75	27	180	41.28
wattled plover	2	80	50	110	42.43
**plover and courser totals**	55	61.89	4	180	37.61

Group averages are means of species-means.

**Table 2:** Table of colour differences in JNDs (“just noticeable differences”).

Species	Best JND match			Mean JND match		
	Ferret	Human	Peafowl	Ferret	Human	Peafowl
Plover & Courser eggs						
three-banded plover	0.23 ± 0.12	0.74 ± 0.48	1.49 ± 0.90	1.24 ± 0.47	1.23 ± 0.73	4.25 ± 2.06
Temminck's courser	0.33 ± 0.26	1.20 ± 0.44	1.61 ± 1.45	0.81 ± 0.39	1.44 ± 0.40	3.22 ± 1.59
bronze-winged courser	0.26 ± 0.23	0.45 ± 0.32	1.22 ± 0.91	1.41 ± 0.53	1.21 ± 0.51	3.75 ± 1.16
three-banded courser	0.22 ± 0.18	0.42 ± 0.15	1.11 ± 0.53	1.08 ± 0.16	0.84 ± 0.25	2.86 ± 0.31
crowned plover	0.20 ± 0.19	0.50 ± 0.31	1.17 ± 1.10	0.73 ± 0.35	0.77 ± 0.39	2.73 ± 1.24
wattled plover	0.20 ± 0.23	0.47 ± 0.36	2.00 ± 1.66	1.10 ± 0.43	1.00 ± 0.44	3.43 ± 1.94
All	0.23 ± 0.21	0.57 ± 0.40	1.31 ± 1.13	0.97 ± 0.50	0.99 ± 0.49	3.16 ± 1.40
Nightjar eggs						
Mozambique nightjar	0.25 ± 0.43	0.99 ± 0.63	2.05 ± 1.34	1.08 ± 0.57	1.49 ± 0.78	4.31 ± 1.92
fiery-necked nightjar	0.11 ± 0.10	0.36 ± 0.24	1.49 ± 1.00	1.35 ± 0.52	1.20 ± 0.53	3.97 ± 1.61
pennant-winged nightjar	0.22 ± 0.20	1.07 ± 0.67	2.93 ± 3.16	1.17 ± 0.33	1.64 ± 0.60	5.47 ± 3.44
All	0.19 ± 0.31	0.72 ± 0.59	1.90 ± 1.57	1.21 ± 0.54	1.37 ± 0.67	4.29 ± 2.05
Nightjar adults						
Mozambique nightjar	0.14 ± 0.17	0.31 ± 0.30	2.23 ± 3.56	0.84 ± 0.29	0.93 ± 0.48	4.93 ± 3.35
fiery-necked nightjar	0.11 ± 0.09	0.30 ± 0.23	2.72 ± 4.57	1.23 ± 0.49	0.83 ± 0.43	6.10 ± 5.55
pennant-winged nightjar	0.12 ± 0.13	0.30 ± 0.39	1.72 ± 1.12	1.14 ± 0.62	1.24 ± 0.65	4.66 ± 1.35
All	0.12 ± 0.14	0.30 ± 0.28	2.38 ± 3.86	1.03 ± 0.46	0.92 ± 0.49	5.40 ± 4.32

Best JND matches show the mean difference between the main egg or adult plumage colour and the closest colour found in the background, whereas Mean JND match is the difference between the egg or adult plumage and all of the background colours. Egg colour values were measured under controlled lighting conditions, whereas adult values were compared within the same *in situ* photograph. Values are means for each group ± 1 standard deviation.
